# Obtaining the necessary molybdenum cofactor for sulfite oxidase activity in the nematode *Caenorhabditis elegans* surprisingly involves a dietary source

**DOI:** 10.1016/j.jbc.2022.102736

**Published:** 2022-11-22

**Authors:** Kevin D. Oliphant, Robin R. Fettig, Jennifer Snoozy, Ralf R. Mendel, Kurt Warnhoff

**Affiliations:** 1 Department of Plant Biology, Braunschweig University of Technology, Braunschweig, Germany; 2 Pediatrics and Rare Diseases Group, Sanford Research, Sioux Falls, South Dakota, USA; 3 Department of Basic Biomedical Sciences, Sanford School of Medicine, University of South Dakota, Vermillion, South Dakota, USA; 4 Department of Pediatrics, Sanford School of Medicine, University of South Dakota, Sioux Falls, South Dakota, USA

**Keywords:** *Caenorhabditis elegans*, molybdenum, diet, genetics, inborn error of metabolism, cPMP, cyclic pyranopterin monophosphate, MPT, molybdopterin, NGM, nematode growth media, Moco, molybdenum cofactor

## Abstract

Molybdenum cofactor (Moco) is a prosthetic group necessary for the activity of four unique enzymes, including the essential sulfite oxidase (SUOX-1). Moco is required for life; humans with inactivating mutations in the genes encoding Moco-biosynthetic enzymes display Moco deficiency, a rare and lethal inborn error of metabolism. Despite its importance to human health, little is known about how Moco moves among and between cells, tissues, and organisms. The prevailing view is that cells that require Moco must synthesize Moco *de novo.* Although, the nematode *Caenorhabditis elegans* appears to be an exception to this rule and has emerged as a valuable system for understanding fundamental Moco biology. *C. elegans* has the seemingly unique capacity to both synthesize its own Moco as well as acquire Moco from its microbial diet. However, the relative contribution of Moco from the diet or endogenous synthesis has not been rigorously evaluated or quantified biochemically. We genetically removed dietary or endogenous Moco sources in *C. elegans* and biochemically determined their impact on animal Moco content and SUOX-1 activity. We demonstrate that dietary Moco deficiency dramatically reduces both animal Moco content and SUOX-1 activity. Furthermore, these biochemical deficiencies have physiological consequences; we show that dietary Moco deficiency alone causes sensitivity to sulfite, the toxic substrate of SUOX-1. Altogether, this work establishes the biochemical consequences of depleting dietary Moco or endogenous Moco synthesis in *C. elegans* and quantifies the surprising contribution of the diet to maintaining Moco homeostasis in *C. elegans.*

Molybdenum cofactor (Moco) is a prosthetic group that was present in the last universal common ancestor and continues to be synthesized in all domains of life by a conserved biosynthetic pathway (Fig. 1) (1, 2). Moco supports the activity of four animal enzymes: sulfite oxidase, xanthine dehydrogenase, aldehyde oxidase, and mitochondrial amidoxime reducing component (3, 4). Thus, Moco is essential to support core metabolic pathways such as sulfur amino acid and purine metabolism.

**Figure 1:**
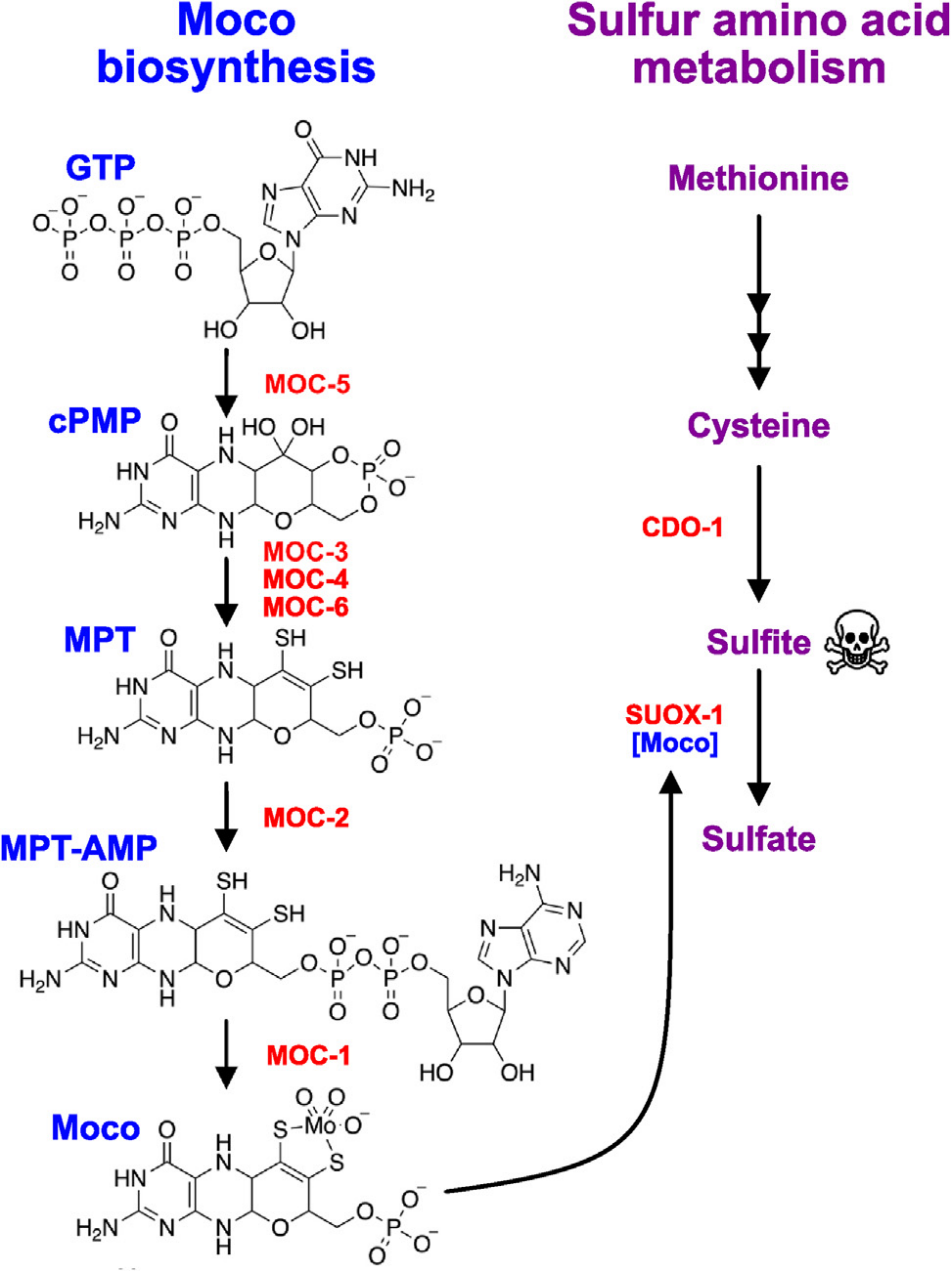
**Intersection of Moco biosynthesis and sulfur amino acid metabolism in *C. elegans*.** The *C. elegans* Moco biosynthetic and sulfur amino acid metabolism (simplified) pathways are displayed (enzymes, *red*). Structures of the following Moco-biosynthetic intermediates are displayed: guanosine triphosphate (GTP), cyclic pyranopterin monophosphate (cPMP), molybdopterin (MPT), molybdopterin adenosine monophosphate (MPT-AMP), and molybdenum cofactor (Moco). Moco, molybdenum cofactor.

Moco was initially revealed in the 1960s through elegant genetic studies in the fungus *Aspergillus nidulans* (5, 6). Chemical mutagenesis screens were performed, leading to the isolation of mutants defective in both nitrate reductase and xanthine dehydrogenase activity. These mutants affected multiple genetic loci outlining the genes necessary for synthesizing CNX, a cofactor common to nitrate reductase and xanthine dehydrogenase (6). CNX is now known as Moco.

Paralleling the genetic understanding of Moco deficiency in *A. nidulans*, human Moco deficiency was first documented in 1978 as a combined deficiency of xanthine dehydrogenase and sulfite oxidase, two Moco-requiring enzymes (7). Causative genetic lesions in human patients affected the orthologous biosynthetic pathway identified initially in *A. nidulans.* Patients presented with a severe range of symptoms as neonates, including difficulty feeding, neurological dysfunction, and eye lens dislocation. Symptoms typically appear within the first week of life and patients generally die within 3 years of birth (8). Excitingly, a therapy has been developed to treat a subset of patients suffering from Moco deficiency (9). Patients with defects in cyclic pyranopterin monophosphate (cPMP) synthesis can receive supplemental cPMP, which is converted to Moco by the healthy downstream Moco biosynthetic machinery. Unfortunately, cPMP therapy is only effective for patients with mutations in the first step in Moco synthesis. Supplementation with mature Moco seems a logical therapy to treat all forms of Moco deficiency. However, the instability and sensitivity of Moco to oxidation have proven experimentally limiting and precluded it from therapeutic consideration. Moco is a unique pterin and its chemical nature was ultimately resolved by studying its stable degradation products, one of which is known as “FormA” (10, 11).

Given its unstable nature, very little is known about how Moco moves within and between cells. Most Moco-requiring cells are believed to synthesize Moco *de novo.* However, recent advances in the nematode *Caenorhabditis elegans* have demonstrated that Moco can move not only between cells and tissues but between disparate organisms (12, 13, 14). Unlike other animals studied, *C. elegans* with null mutations in genes encoding the Moco-biosynthetic enzymes are viable and fertile. These genes are termed *moc* in *C. elegans* for molybdenum cofactor biosynthesis. *moc* mutant animals are viable because *C. elegans* stably acquires mature Moco from its bacterial diet. This dietary Moco is stabilized by Moco-binding/using proteins and is transported by unknown mechanisms throughout the *C. elegans* animal to support the activity of its Moco-requiring enzymes (13). Unsurprisingly, Moco is essential for *C. elegans* viability: animals lacking both endogenous Moco synthesis and dietary Moco arrest during larval development and die. This lethality is caused by the inactivity of sulfite oxidase (SUOX-1), a Moco-requiring enzyme that oxidizes the lethal toxin sulfite to sulfate (14). Sulfite oxidase is also essential in humans. Patients with inactivating mutations in sulfite oxidase develop clinical and biochemical features that mirror Moco deficiency, highlighting that SUOX-1 is likely the key Moco-requiring enzyme supporting animal life (7, 15).

We reason that dietary Moco must enter the *C. elegans* animal through the intestine. However, the primary tissue responsible for Moco synthesis and use seems to be the hypodermis, a distal tissue. Tissue-specific expression of the *moc-1* gene, encoding a Moco biosynthesis enzyme (Fig. 1), strongly rescues *moc-1(-)* null mutant phenotypes when transcribed from a hypodermal-specific promoter. *moc-1* expression in muscle cells also rescues *moc-1(-)* mutant phenotypes, albeit to a lesser extent (14). Furthermore, only hypodermal expression of *suox-1* was sufficient to rescue the lethality of a *suox-1(-)* null mutant animal (14). These results suggest Moco is both synthesized and acting primarily in the *C. elegans* hypodermis. Considering these findings, the functionality of dietary Moco is even more impressive: dietary Moco must travel from the environment, through the intestine, and be distributed to the hypodermis where it ultimately supports SUOX-1 activity. This journey likely involves the stable crossing of multiple cell membranes *via* an unknown mechanism.

Here, we adapt biochemical protocols to quantify the contribution of dietary Moco *versus* endogenously synthesized Moco. We use genetic strategies to remove either source of Moco and evaluate the impact on (i) the activity of the Moco-requiring SUOX-1 enzyme and (ii) *C. elegans* Moco content found in crude *C. elegans* extracts. Furthermore, we evaluate the physiological impact of removing dietary Moco *versus* endogenously synthesized Moco by examining growth and development under pharmacological and genetic conditions where sulfite concentrations are toxic to animal growth. We demonstrate that dietary Moco and endogenously synthesized Moco are nonredundantly promoting Moco accumulation and SUOX-1 activity. Furthermore, removing either source of Moco causes sulfite sensitivity, indicating that both dietary Moco and endogenous Moco synthesis are required to promote optimal *C. elegans* fitness.

## Results

### Biochemical quantification of *C. elegans* SUOX-1 activity and Moco content

To biochemically evaluate *C. elegans* Moco homeostasis, we generated crude extracts from large cultures of young adult animals. Animals were cultured on solid agar media seeded with a monoculture of *Escherichia coli*, the food source of *C. elegans* cultivated in the laboratory*.* Young adult animals were collected, extensively washed to remove *E. coli* cells, and then snap-frozen in liquid nitrogen. Samples were subsequently lysed using a bead beater, and protein concentrations were determined *via* Bradford protein assay to allow for downstream normalization.

These crude extracts were then used to determine SUOX-1 activity and Moco content. We first analyzed samples that we anticipated would have maximal and minimal SUOX-1 activity and Moco content to validate these approaches. We reasoned that WT *C. elegans* fed WT (Moco+) *E. coli* would have high levels of SUOX-1 activity and Moco as both endogenous Moco synthesis and dietary Moco uptake are functional in this context. In contrast, we used the *moc-4; cdo-1* double mutant *C. elegans* fed *ΔmoaA* mutant (Moco-) *E. coli* as the sample where we anticipated little to no SUOX-1 activity or Moco content (14). To validate that WT *E. coli* are Moco replete and *ΔmoaA* mutant *E. coli* are Moco deficient, we performed HPLC analyses and observed Moco content as detected by the fluorescent derivative dephospho-FormA (_dp_FormA) (10). Indeed, we detected a strong _dp_FormA peak when analyzing WT *E. coli* and did not detect a peak in *ΔmoaA* mutant *E. coli* (Fig. S1). *moc-4; cdo-1* double mutant *C. elegans* cannot synthesize their own Moco due to the null mutation in the *moc-4* gene that encodes molybdopterin synthase (Fig. 1). *moc-4* mutant animals typically display 100% penetrant larval arrest and death when cultured on Moco- *E. coli.* However, this lethality can be suppressed by mutations in *cdo-1*, which encodes cysteine dioxygenase (14). CDO-1 is a critical enzyme in the catabolism of sulfur amino acids and promotes the production of sulfites, the critical toxin when Moco is absent (Fig. 1) (14, 16, 17, 18). *cdo-1* inactivation prevents the accumulation of sulfites and allows *C. elegans* animals to grow in the absence of endogenous and dietary Moco sources. Thus, we speculated that *moc-4; cdo-1* double mutant animals cultured on Moco- *E. coli* would be completely Moco deficient and lack SUOX-1 activity.

**Figure 2:**
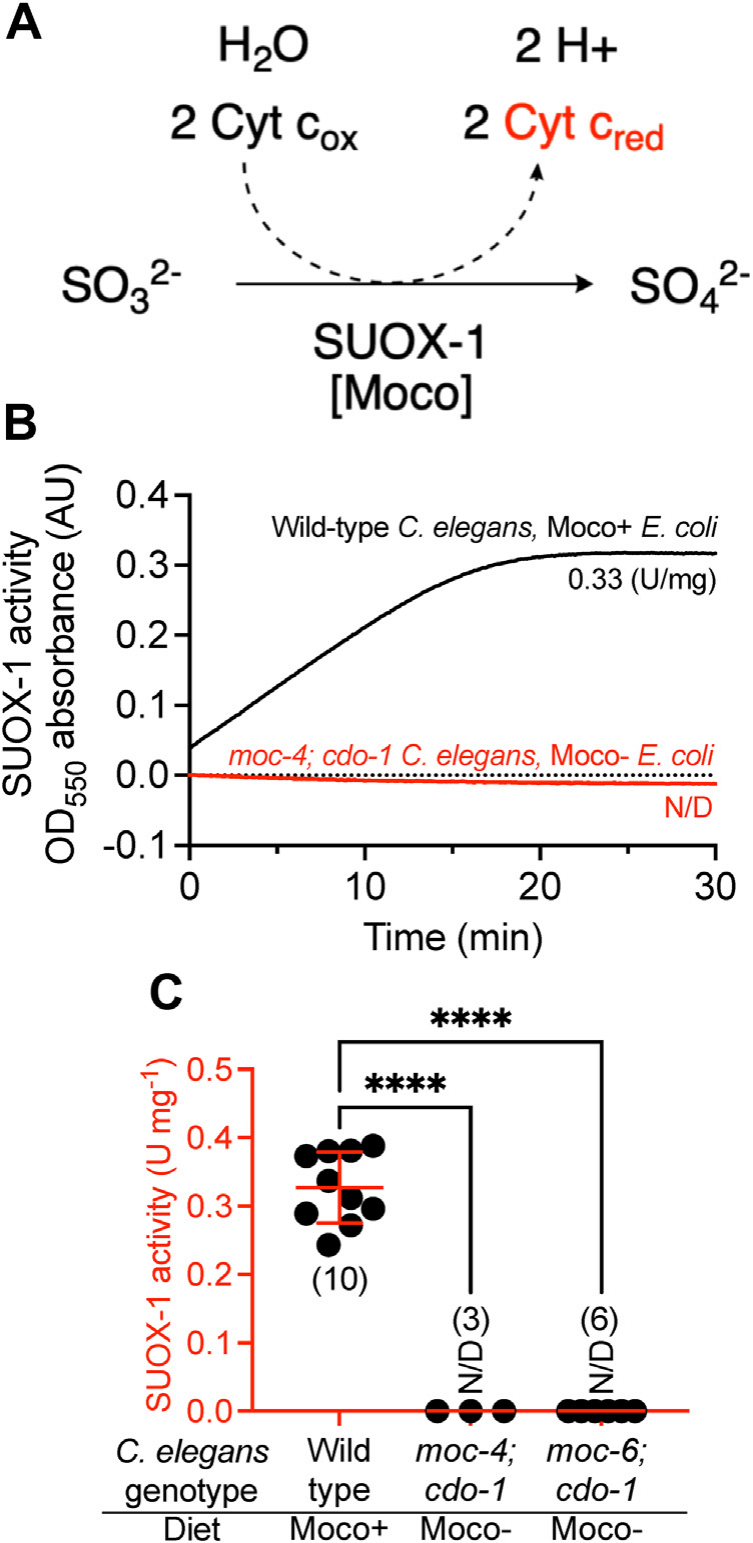
**Quantifying SUOX-1 activity in crude extracts from *C. elegans.****A*, simplified reaction mechanism for SUOX-1 by which sulfite (SO_3_^2-^) is oxidized to sulfate (SO_4_^2-^). Sulfite-dependent SUOX-1 activity is detected *via* the concomitant reduction of cytochrome c. *B*, SUOX-1 activity was detected in crude extracts from WT *C. elegans* fed WT (Moco+) *E. coli* (*black*) or *moc-4(ok2571)*; *cdo-1(mg622)* mutant *C. elegans* fed *ΔmoaA* (Moco-) *E. coli* (*red*). Units of SUOX-1 activity per milligram of protein are displayed. *C*, SUOX-1 activity is displayed for WT and mutant *C. elegans* fed either WT (Moco+) or *ΔmoaA* (Moco-) *E. coli.* All biological replicates, the sample size, mean, and SD are displayed for each condition. N/D indicates no SUOX-1 activity was detected in any sample. ∗∗∗∗*p* < 0.0001, ordinary one-way ANOVA with Dunnet’s post hoc analysis. Note, the WT data in Figure 2*C* are also displayed in Figure 4, Figure 5, Figure 6*A*, to allow for appropriate graphical comparisons of data. Moco, molybdenum cofactor.

To determine SUOX-1 activity of these samples, we modified an assay based on a photometrically quantifiable reduction of cytochrome c (Fig. 2*A*) (19). In this reaction, sulfite is converted to sulfate, and cytochrome c acts as an electron donor. The resulting reduced form of cytochrome c has an increased absorption at *A*_550_, which we detect and quantify. Aligning with our expectations, we readily detected SUOX-1 activity from extracts of WT *C. elegans* fed Moco+ *E. coli* (0.33 U/mg), while we were unable to detect SUOX-1 activity from *moc-4; cdo-1* double mutant animals fed Moco- *E. coli* (Fig. 2, *B* and *C*)*. moc-6* encodes another component of the Moco-biosynthetic machinery and is necessary for *C. elegans* Moco synthesis (Fig. 1) (12). Supporting our findings with *moc-4; cdo-1* animals, we were unable to detect SUOX-1 activity in crude extracts from *moc-6; cdo-1* double mutant animals fed Moco- *E. coli* (Fig. 2*C*)*.* These results align well with prior genetic data and validate this assay to quantify SUOX-1 activity from crude *C. elegans* extracts (14).

**Figure 3:**
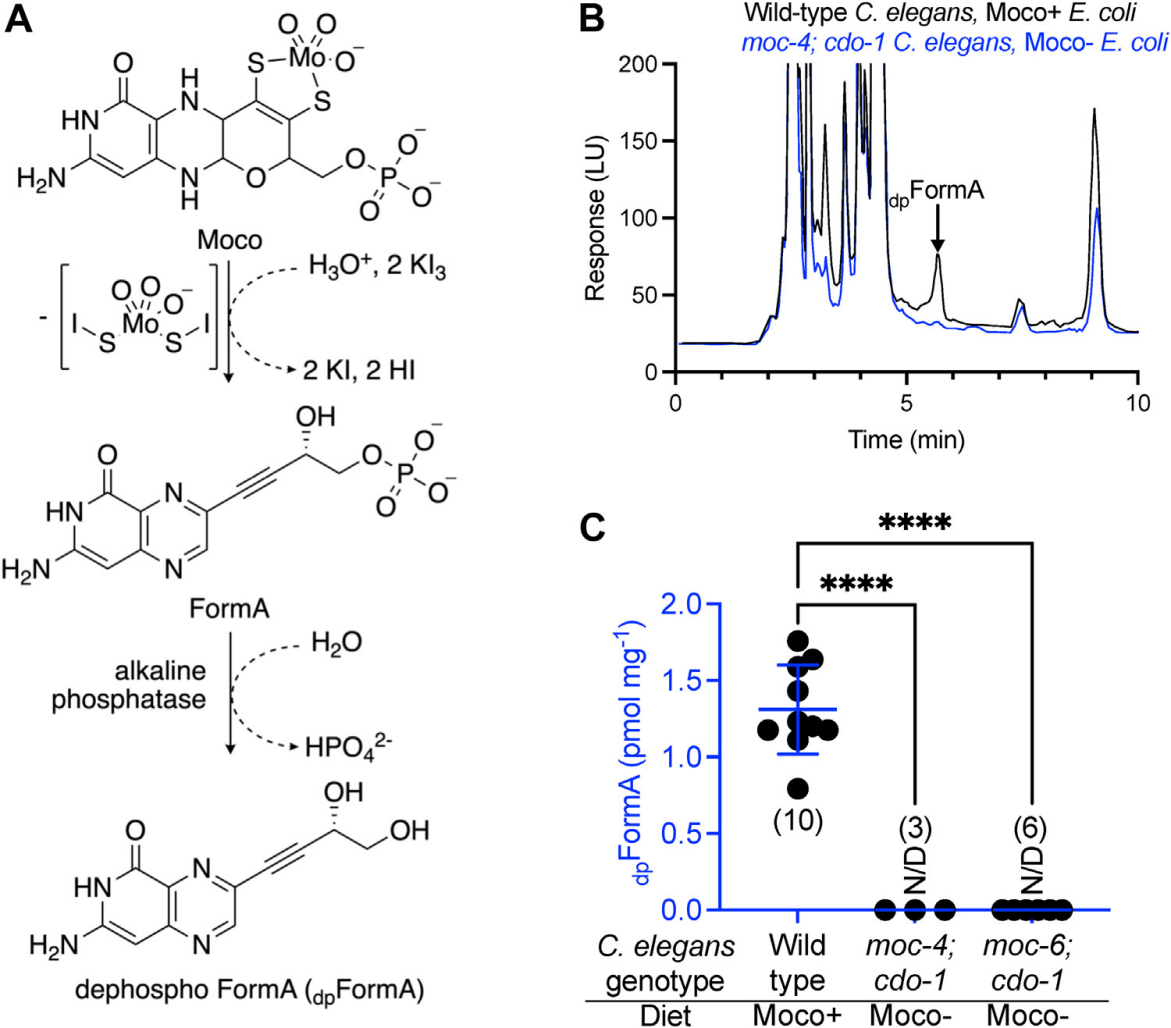
**Quantifying Moco content in crude extracts from *C. elegans.****A*, a schematic of the conversion of Moco to dephospho-FormA (_dp_FormA) in the presence of an acidic iodine environment and treatment with alkaline phosphatase. *B*, HPLC measurements of Moco-derived _dp_FormA from crude extracts of WT *C. elegans* fed WT (Moco+) *E. coli* (*black*) or *moc-4(ok2571)*; *cdo-1(mg622)* mutant *C. elegans* fed *ΔmoaA* (Moco-) *E. coli* (*blue*). The _dp_FormA peak is indicated (*black arrow*). *C*, _dp_FormA content is displayed for WT, and mutant *C. elegans* fed either WT (Moco+) or *ΔmoaA* (Moco-) *E. coli.* All biological replicates, the sample size, mean, and SD are displayed for each condition. N/D indicates no _dp_FormA was detected in any sample. ∗∗∗∗*p* < 0.0001, ordinary one-way ANOVA with Dunnet’s post hoc analysis. Note, the WT data in Figure 3*C* are also displayed in Figures 4*B* and 5*B* to allow for appropriate graphical comparisons of data. Moco, molybdenum cofactor.

To determine Moco content of these same samples, crude extracts were oxidized and subsequently treated with alkaline phosphatase. Iodine-dependent oxidation and dephosphorylation convert highly unstable Moco and molybdopterin (MPT) to the stable and fluorescent derivative, dephospho-FormA (_dp_FormA) (Fig. 3*A*) (10). Oxidized metabolites were then analyzed *via* HPLC (20). _dp_FormA was detected with an average elution time of 5:40 min and quantified using established methods and standards (21). Supporting our genetic hypotheses, we readily detected _dp_FormA in crude extracts of WT *C. elegans* fed Moco+ *E. coli* (1.3 pmol/mg) and were not able to detect Moco in *moc-4; cdo-1* mutant animals fed Moco- *E. coli* (Fig. 3, *B* and *C*)*.* We also analyzed crude extracts from *moc-6; cdo-1* double mutant animals fed Moco- *E. coli* and were unable to detect Moco-derived _dp_FormA (Fig. 3*C*). To further validate that the peak highlighted in Figure 3*B* is _dp_FormA, the same samples were prepped and analyzed without alkaline phosphatase treatment. To be detected on a reversed-phase column, FormA must be dephosphorylated or it will not interact with the solid phase and elute in the void volume. Without alkaline phosphatase treatment, we did not see the highlighted peak in any of our samples, supporting our claim that this peak is _dp_FormA (Fig. S2). These results align well with expectations and validate this assay for quantifying Moco content from crude *C. elegans* extracts.

### Dietary Moco is necessary to promote SUOX-1 activity and Moco content in *C. elegans*

Having established new methodology for quantifying SUOX-1 activity and Moco content in *C. elegans*, we sought to address some fundamental questions regarding Moco homeostasis*.* Principally, how much of the total Moco content of *C. elegans* is derived from the diet as compared to endogenous synthesis? To address this question, we generated *C. elegans* samples where we deprived the animals of either (i) dietary Moco (WT *C. elegans* fed Moco- *E. coli*) or (ii) endogenous Moco synthesis (*moc-4* mutant *C. elegans* fed Moco+ *E. coli*). Extracts were generated from these samples, and SUOX-1 activity and Moco content were determined.

**Figure 4:**
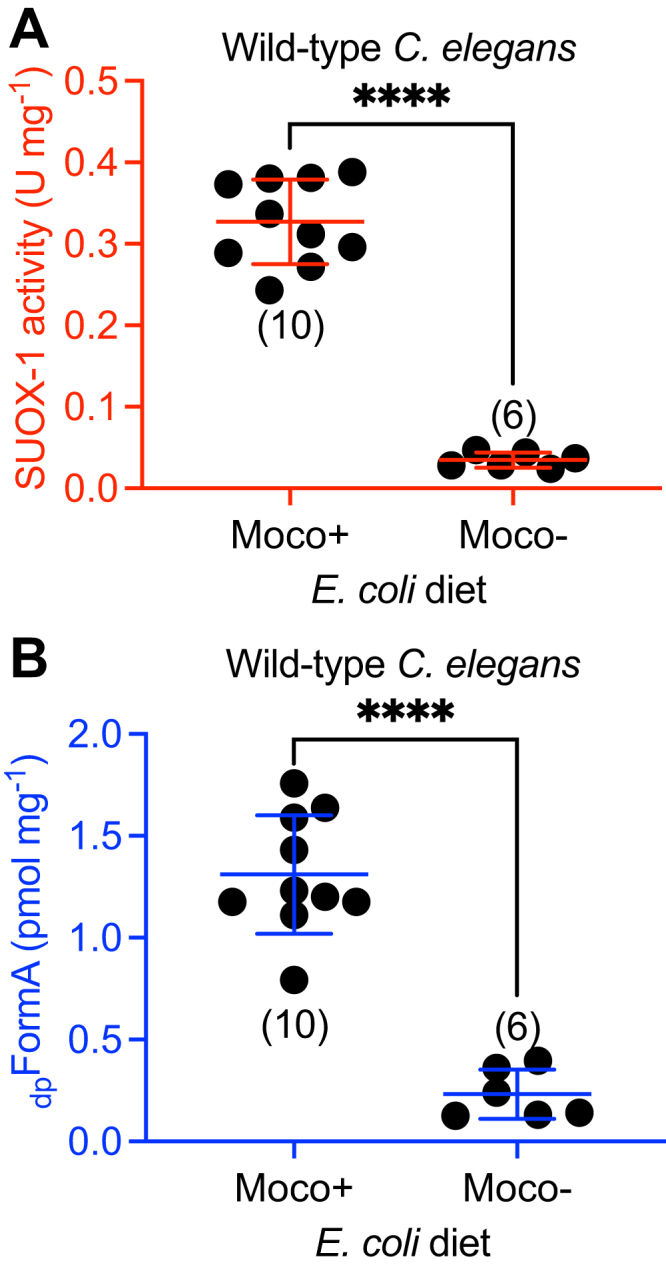
**Dietary Moco is necessary to promote SUOX-1 activity and Moco accumulation.***A*, SUOX-1 activity and (*B*) _dp_FormA content are displayed for WT *C. elegans* fed either WT (Moco+) or *ΔmoaA* (Moco-) *E. coli.* All biological replicates, the sample size, mean, and SD are displayed for each condition. ∗∗∗∗*p* < 0.0001, Welch’s *t* test. Moco, molybdenum cofactor.

WT *C. elegans* fed Moco- *E. coli* displayed only 11% SUOX-1 activity compared to WT *C. elegans* fed a Moco+ diet (Fig. 4*A*). This surprising result demonstrates that dietary Moco is necessary to promote SUOX-1 activity in WT *C. elegans*. Similar results were observed when we analyzed the same samples for Moco content. WT *C. elegans* fed Moco- *E. coli* displayed only 18% Moco content when compared to the same animals fed a Moco+ diet (Fig. 4*B*). These data demonstrate that dietary Moco is necessary to promote Moco accumulation in WT *C. elegans.*

### *C. elegans* Moco biosynthesis is necessary to promote SUOX-1 activity and Moco content

**Figure 5:**
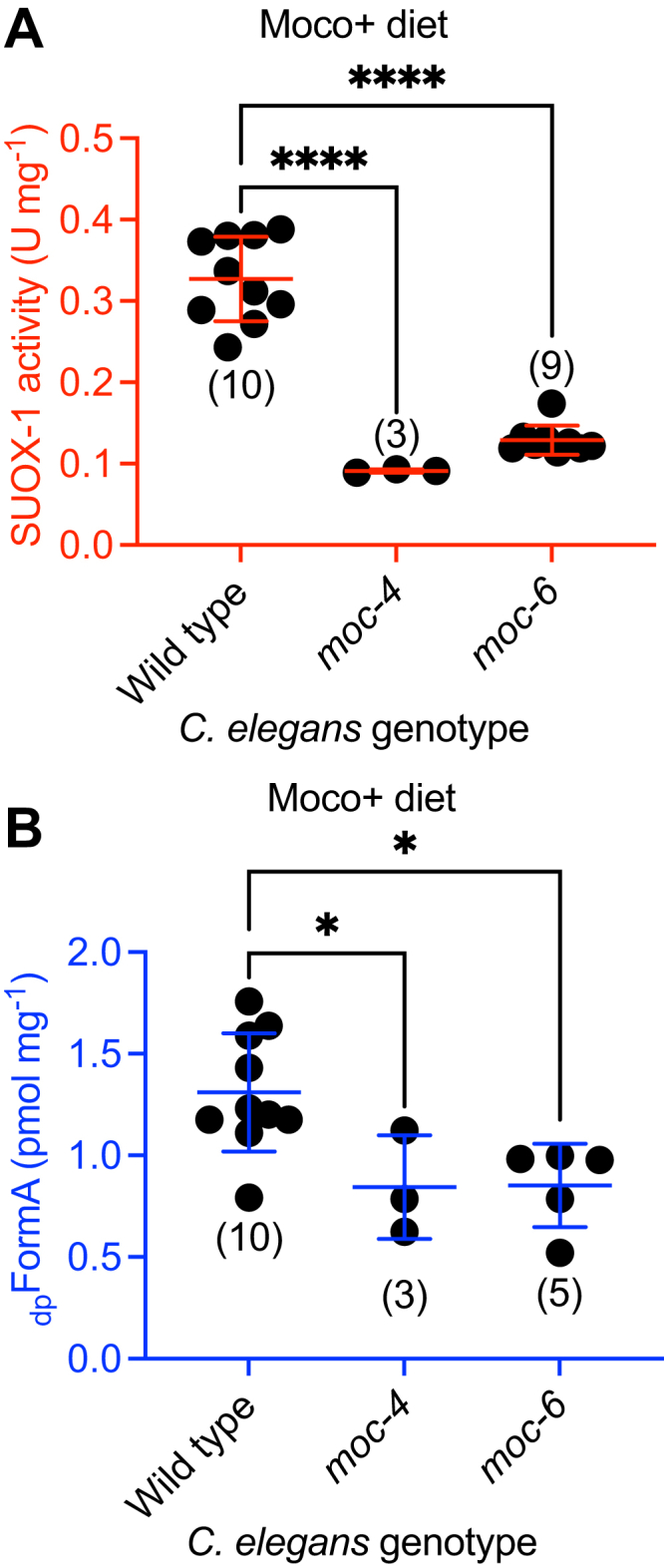
**Endogenous Moco synthesis is necessary to promote SUOX-1 activity and Moco accumulation.***A*, SUOX-1 activity and (*B*) _dp_FormA content are displayed for WT, *moc-4*, and *moc-6* mutant *C. elegans* fed WT (Moco+) *E. coli.* All biological replicates, the sample size, mean, and SD are displayed for each condition. ∗*p* < 0.05 and ∗∗∗∗*p* < 0.0001, ordinary one-way ANOVA with Dunnet’s post hoc analysis. Moco, molybdenum cofactor.

Given the critical role of dietary Moco in supporting SUOX-1 activity and animal Moco content, we wondered about the relative importance of endogenous Moco synthesis. To evaluate this, we cultured *moc-4* mutant *C. elegans* on Moco+ *E. coli*, restricting the animal’s Moco source to the diet. We generated crude extracts from these samples and determined SUOX-1 activity and Moco content. *moc-4* mutant animals had 28% of the SUOX-1 activity displayed by their WT counterparts and 64% of the Moco content (Fig. 5). We saw similar results when evaluating *moc-6* mutant animals fed Moco+ *E. coli*, which displayed 39% of WT SUOX-1 activity and 65% of Moco content (Fig. 5). These data demonstrate that endogenous Moco synthesis is necessary to promote SUOX-1 activity and Moco accumulation in *C. elegans.*

### The *C. elegans suox-1(gk738847)* mutation causes reduced SUOX-1 activity and sulfite sensitivity

**Figure 6:**
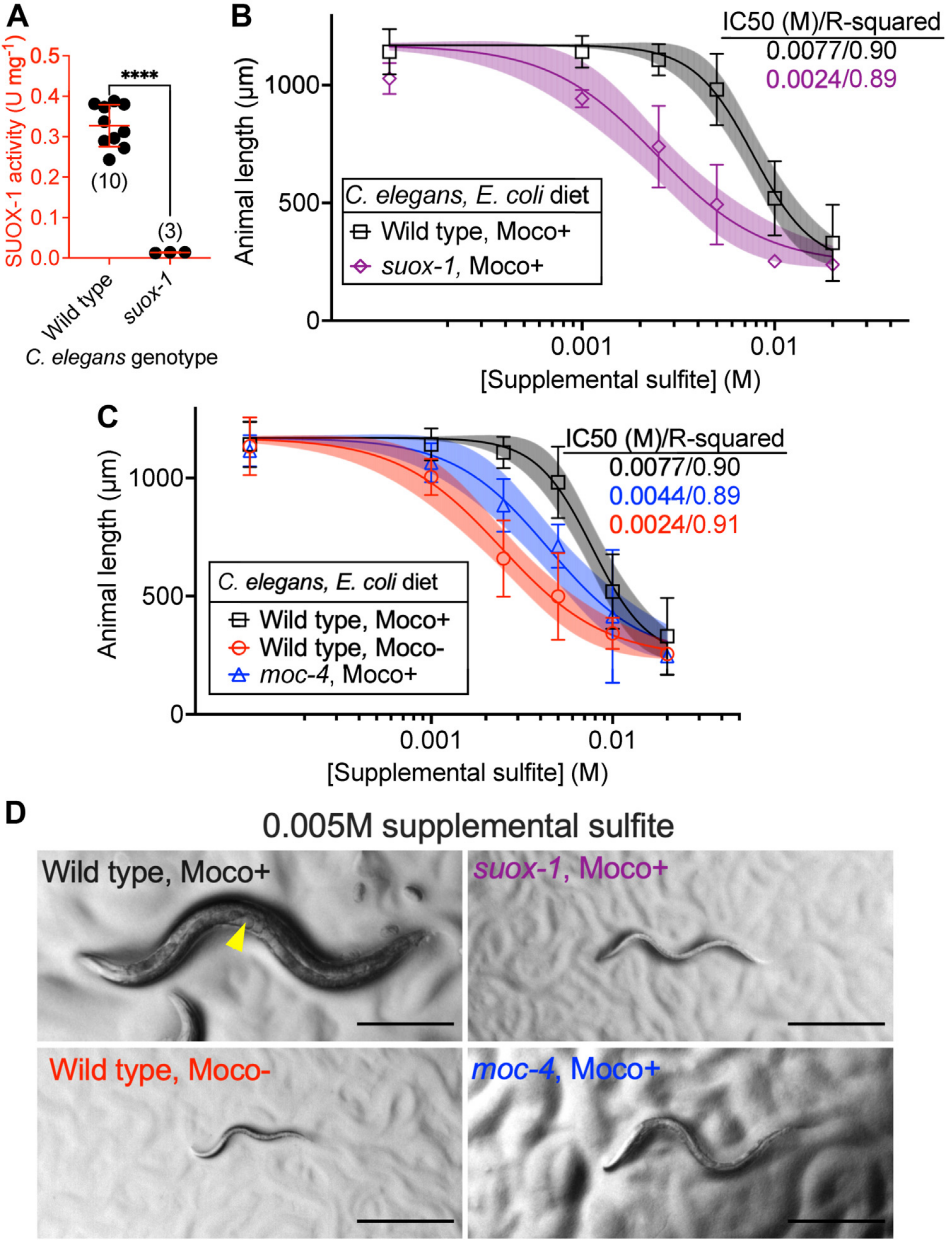
**Dietary Moco and endogenous Moco synthesis are nonredundantly required for sulfite tolerance.***A*, SUOX-1 activity is displayed for WT and *suox-1(gk738847)* mutant *C. elegans* fed WT (Moco+) *E. coli.* All biological replicates, the sample size, mean, and SD are displayed for each condition. ∗∗∗∗*p* < 0.0001, Welch’s *t* test. *B* and *C*, WT and mutant *C. elegans* were synchronized at L1 stage and cultured on WT (Moco+) or *ΔmoaA* mutant (Moco-) *E. coli* supplemented with various concentrations of sulfite (0, 0.0001, 0.001, 0.0025, 0.005, 0.01, and 0.02M). Animal length was measured after 72 h of growth at 20 °C. Data points represent the average of 3 (*suox-1,* Moco+ (*purple*) and *moc-4,* Moco+ (*blue*)) or 4 (WT, Moco+ (*black*) and WT, Moco- (*red*)) biological replicates. For each biological replicate, 15 or more individual *C. elegans* animals were imaged and measured at each sulfite concentration. Data points indicate mean and error bars display SD. IC_50_ was calculated by nonlinear regression analyses and shading indicates the 95% confidence interval. IC_50_ and R-squared values are displayed. Note that the data displayed for WT *C. elegans* fed Moco+ *E. coli* in Figure 6*B* are identical to the same control displayed in Figure 6*C*. *D*, Representative individuals are displayed at the critical 0.005M supplemental sulfite concentration. The scale bar represents 250 μm. *Yellow* arrowhead indicates embryos in the uterus of the gravid adult animal. Moco, molybdenum cofactor.

SUOX-1 is an essential enzyme in both *C. elegans* and humans (14, 15). To evaluate the role of *suox-1* in *C. elegans* biology, we use the hypomorphic *suox-1(gk738847)* allele as a genetic tool as it reduces *suox-1* function but does not cause overt developmental phenotypes (13, 14, 22). *gk738847* is a missense mutation resulting in the amino acid substitution D391N. Aspartic acid 391 is highly conserved from *C. elegans* to humans (23). To characterize the impact of the D391N substitution to SUOX-1 activity, we prepared crude extracts from young adult WT and *suox-1(gk738847)* animals cultured on Moco+ *E. coli.* SUOX-1 activity of these extracts was then evaluated. Our data demonstrate that *suox-1(gk738847)* mutant animals displayed 4% SUOX-1 activity compared to their WT counterparts (Fig. 6*A*). Despite this dramatic reduction in SUOX-1 activity, *suox-1(gk738847)* mutant animals appear superficially WT when cultured under standard laboratory conditions. To probe for fitness defects caused by reduced SUOX-1 activity, we cultured WT and *suox-1(gk738847)* animals on Moco+ bacteria exposed to various concentrations of supplemental sulfite. WT *C. elegans* fed Moco+ bacteria tolerate high sulfite well, displaying a IC_50_ of 0.0077M supplemental sulfite. In contrast, *suox-1(gk738847)* mutant animals are sensitive to sulfite displaying an IC_50_ of 0.0024M supplemental sulfite (Fig. 6, *B* and *D*). These results align well with previous studies (14). Taken together, these data biochemically demonstrate the effect of the *suox-1(gk738847)* D391N mutation on the activity of SUOX-1 in crude *C. elegans* extracts. Furthermore, we show that *suox-1* is essential for tolerating high supplemental sulfite and establish a sulfite-sensitivity assay for detecting physiological outcomes of reduced SUOX-1 activity.

### Dietary Moco and endogenous Moco synthesis are nonredundantly required for sulfite tolerance

Our biochemical analyses demonstrate that WT *C. elegans* fed a diet of Moco- *E. coli* show decreased SUOX-1 activity (Fig. 4*A*). Like *suox-1(gk738847)* mutant animals, WT *C. elegans* provided Moco- *E. coli* appear superficially healthy under standard culture conditions (14). We hypothesized that the reductions in SUOX-1 activity caused by a Moco- diet would cause sulfite sensitivity. To test this, we exposed WT *C. elegans* cultured on Moco+ or Moco- *E. coli* to various concentrations of supplemental sulfite and analyzed growth and development. WT *C. elegans* fed Moco+ *E. coli* tolerate sulfite well and display an IC_50_ of 0.0077 M supplemental sulfite. By contrast, WT animals fed Moco- *E. coli* were sensitive to supplemental sulfite, displaying an IC_50_ of 0.0024 M supplemental sulfite (Fig. 6, *C* and *D*). These data demonstrate that dietary Moco is necessary for sulfite tolerance in *C. elegans.*

Given the requirement of dietary Moco to support sulfite tolerance, we wondered if endogenous Moco synthesis would also be essential for this process. To test the role of endogenously synthesized Moco in sulfite tolerance, we analyzed *moc-4* null mutant *C. elegans* that cannot synthesize Moco (14). We exposed *moc-4* mutant *C. elegans* cultured on Moco+ *E. coli* to various concentrations of supplemental sulfite and analyzed their growth and development. When compared to WT animals fed Moco+ bacteria, we found that *moc-4* mutant *C. elegans* grown on Moco+ *E. coli* were sensitive to supplemental sulfite, displaying an IC_50_ of 0.0044M supplemental sulfite (Fig. 6, *C* and *D*). Taken together, these data demonstrate that dietary Moco and endogenous Moco synthesis are nonredundantly required for *C. elegans* to tolerate high environmental sulfite.

### Dietary Moco is essential for the development of *suox-1(gk738847)* mutant *C. elegans*

Our biochemical analyses of Moco content and SUOX-1 activity paired with our pharmacological experiments with supplemental sulfite suggest a model for Moco homeostasis whereby endogenous synthesis and dietary acquisition of Moco are acting nonredundantly to promote healthy Moco content in *C. elegans*, thus promoting SUOX-1 activity. To further test this model, we used the *suox-1(gk738847)* allele as a sensitized genetic background. We observed the development of these mutant animals when we altered (i) dietary Moco availability and (ii) endogenous Moco synthesis.

**Figure 7:**
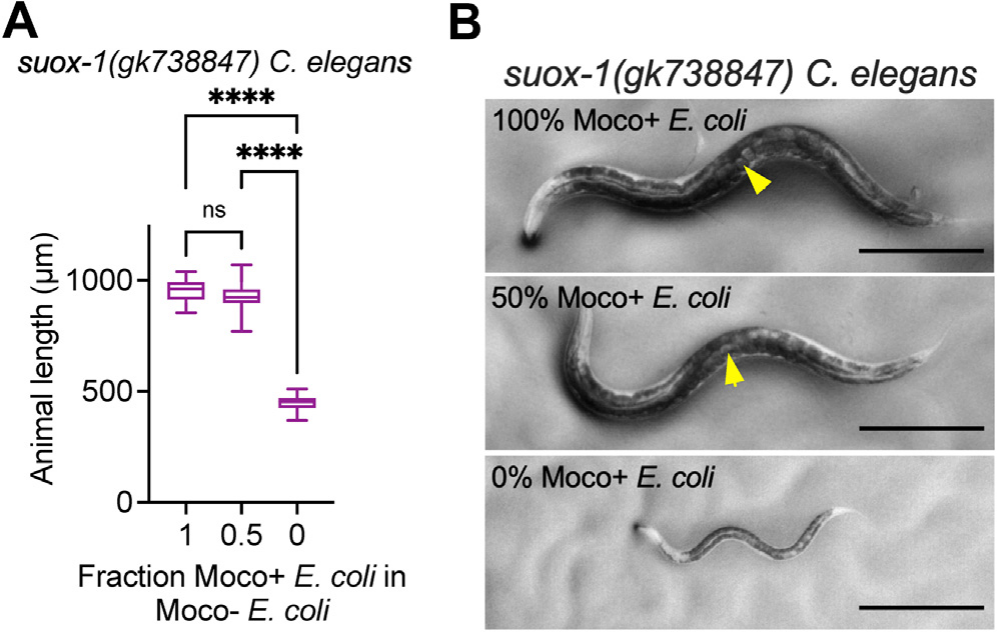
**Dietary Moco is essential for *C. elegans* development when *suox-1* activity is compromised.***A*, *suox-1(gk738847)* mutant *C. elegans* were synchronized at the L1 stage and cultured on mixtures of WT (Moco+) and *ΔmoaA* mutant (Moco-) *E. coli*. Animal length was measured after 72 h of growth at 20 °C. Box plots display the median, upper, and lower quartiles, while whiskers indicate minimum and maximum data points. Sample size is 15 individuals per experiment. ∗∗∗∗*p* < 0.0001, ordinary one-way ANOVA with Tukey’s post hoc analysis. *B*, representative individuals from Figure 7*A* are displayed. The scale bar represents 250 μm. *Yellow* arrowheads indicate embryos in the uterus of gravid adult animals. Moco, molybdenum cofactor.

To test the role dietary Moco plays in supporting the development of *suox-1(gk738847)* mutant *C. elegans*, we cultured *suox-1(gk738847)* mutant animals on Moco+, Moco-, and mixtures of Moco+/Moco- *E. coli. suox-1(gk738847)* mutant animals grew well on Moco+ *E. coli* and 50:50 mixtures of Moco+ and Moco- *E. coli*, reaching fertile adulthood within 72 h of development post first larval (L1) stage (Fig. 7)*.* However, we observe dose-dependent developmental delay in *suox-1(gk738847)* mutant animals at reduced fractions of Moco+ *E. coli* (IC_50_ of 0.06 Moco+ *E. coli* in Moco- *E. coli*, Fig. S3*A*). Furthermore, *suox-1(gk738847)* animals displayed the most substantial reduction in growth and development when cultured on completely Moco- *E. coli*, failing to progress through larval development within the 72 h assay (Figs. 7 and S3*A*)*.* Importantly, WT *C. elegans* reached fertile adulthood in our developmental assays on all mixtures of Moco+ and Moco- *E. coli* (Fig. S3*B*)*.* These data align well with our previous studies and demonstrate that dietary Moco is required for the growth and development of *suox-1(gk738847)* mutant animals (14).

### Endogenous Moco synthesis is essential for the development of *suox-1(gk738847)* mutant *C. elegans*

**Figure 8:**
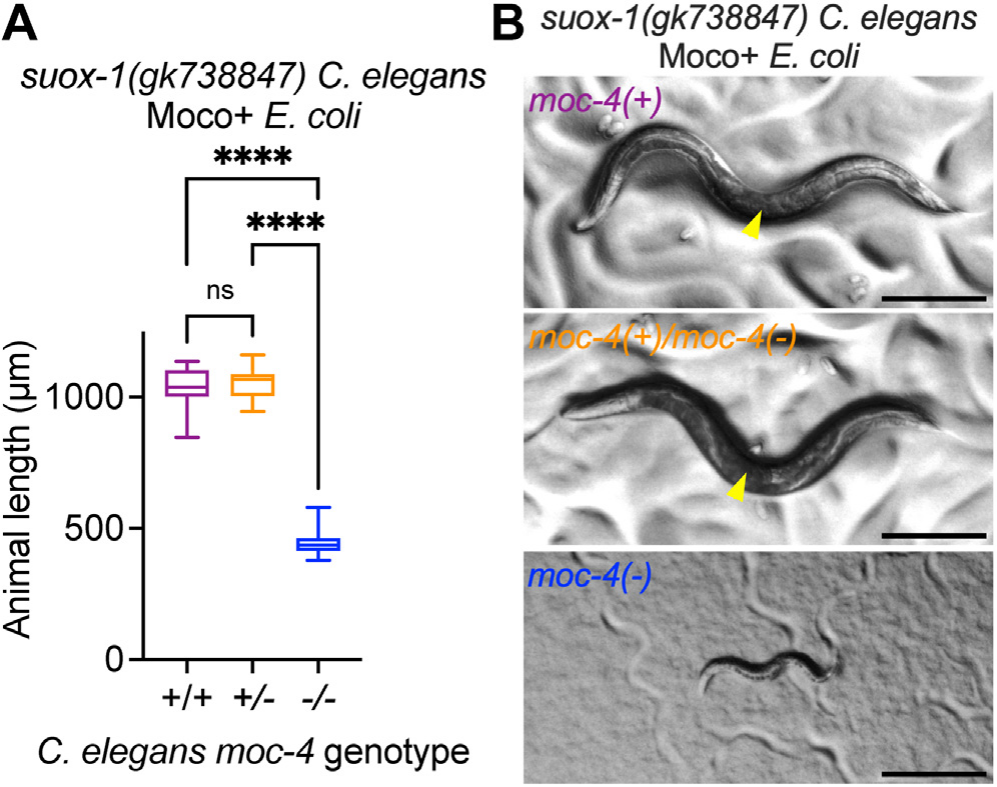
***C. elegans* Moco synthesis is essential when *suox-1* activity is compromised.***A*, *suox-1(gk738847)*, *moc-4(+)/moc-4(ok2571); suox-1(gk738847)*, and *moc-4(ok2571); suox-1(gk738847)* mutant *C. elegans* were synchronized at the L1 stage and cultured on WT (Moco+) *E. coli.* Animal length was measured after 72 h of growth at 20 °C. Box plots display the median, *upper*, and *lower quartiles*, while whiskers indicate minimum and maximum data points. Sample size is 15 individuals per experiment. ∗∗∗∗*p* < 0.0001, ordinary one-way ANOVA, with Tukey’s post hoc analysis. Note, because *moc-4; suox-1* double mutant animals are not viable, they (along with *moc-4(+)/moc-4(-); suox-1* animals) were derived from the balanced strain USD1011 (see Experimental procedures) (24). *B*, representative individuals from Figure 8*A* are displayed. The scale bar represents 250 μm. *Yellow* arrowheads indicate embryos in the uterus of gravid adult animals. Moco, molybdenum cofactor.

**Figure 9:**
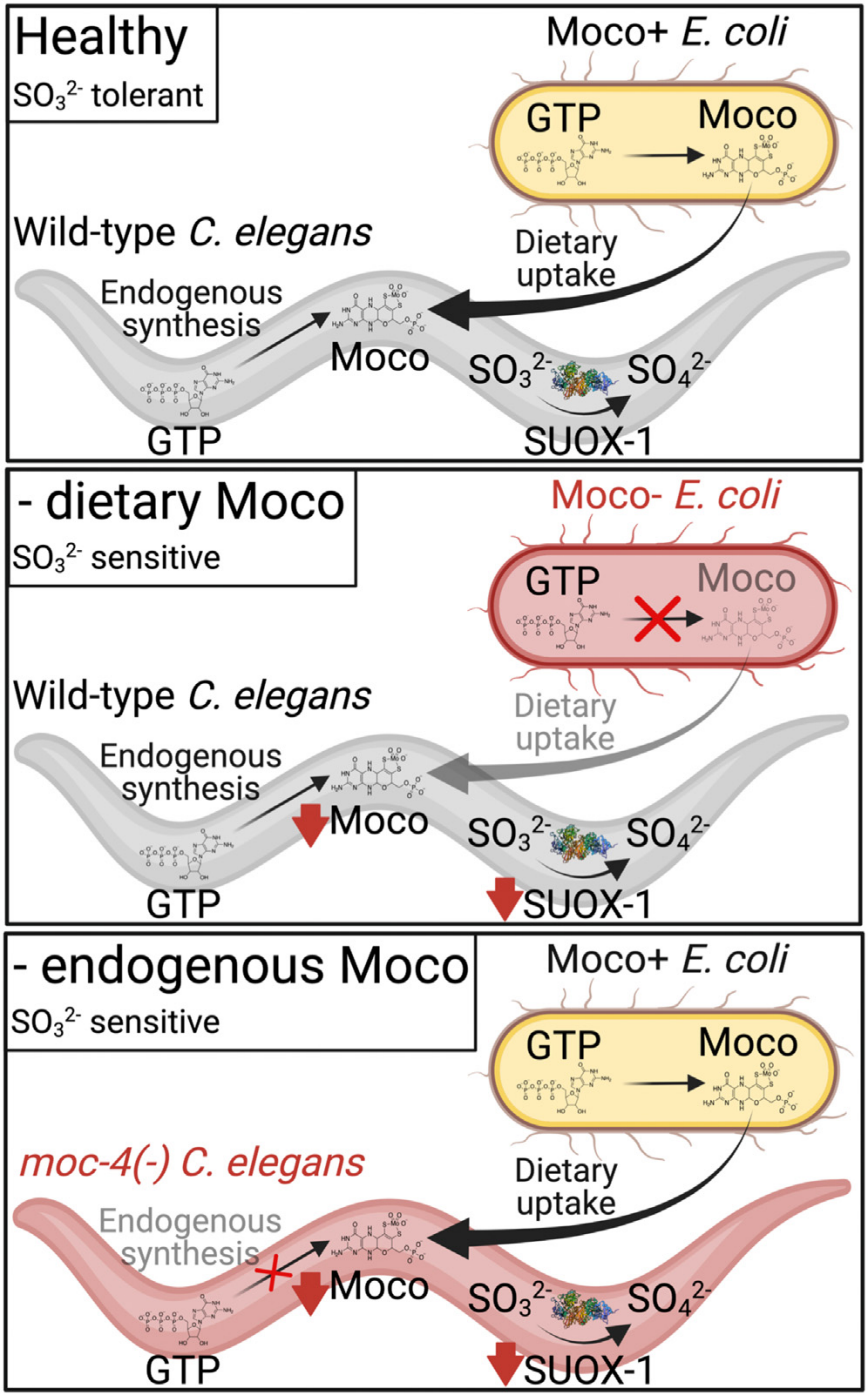
**Moco homeostasis in *C. elegans.*** In healthy control animals, WT *C. elegans* grown on WT *E. coli* (Moco+), SUOX-1 function and Moco content are high, leading to normal development and sulfite tolerance. SUOX-1 activity and Moco content are dramatically reduced when animals are fed a Moco- diet or when the endogenous synthesis of Moco is disrupted. These deficiencies result in sensitivity to sulfite (SO_3_^2−^). Figure created with BioRender. Moco, molybdenum cofactor.

We then sought to test the role of endogenous Moco synthesis in supporting the growth and development of *suox-1(gk738847)* mutant *C. elegans.* To test this, we attempted to engineer double mutant strains of *C. elegans* by combining the *suox-1(gk738847)* allele with various mutations in *C. elegans* Moco-biosynthetic enzymes (*moc-1(ok366)*, *moc-4(ok2571)*, *moc-5(mg589)*, and *moc-6(rae296)*) (12, 14)*.* In our attempts to construct these double mutants, we were unable to isolate viable double mutant strains. Furthermore, dead larvae were observed in all stages of mutant construction where we would have expected the double mutant to emerge. Thus, we hypothesized a synthetic lethal interaction between mutations in *moc* genes and the *suox-1(gk738847)* hypomorphic allele. To test this hypothesis, we constructed a strain where *suox-1(gk738847)* was homozygous and the *moc-4(ok2571)* allele was balanced by the *tmC18* balancer (24). Given this strain, we could synchronize animals at the L1 stage and unequivocally evaluate the growth and development of *moc-4(-); suox-1(gk738847)* and *moc-4(+)/moc-4(-); suox-1(gk738847)* animals and compare their growth to *suox-1(gk738847)* single mutant animals. These mutant *C. elegans* were fed Moco+ *E. coli* throughout the experiment. Consistent with our earlier failed efforts to generate *moc; suox-1(gk738847)* double mutant animals, *moc-4(-); suox-1(gk738847)* double mutant *C. elegans* derived from the balancer strain grew extremely slowly, did not reach fertile adulthood, and displayed larval lethality (Fig. 8). By contrast, *moc-4(+)/moc-4(-); suox-1(gk738847)* and *suox-1(gk738847)* mutant animals grew well, reaching fertile adulthood within 72 h of development post L1 stage (Fig. 8). These data demonstrate that when *suox-1* activity is reduced *via* the *gk738847* mutation, endogenous Moco synthesis is essential. These results support a model whereby dietary Moco and endogenous Moco synthesis are nonredundantly promoting Moco accumulation in *C. elegans.* Increased Moco content is likely directly promoting SUOX-1 activity, which promotes sulfite tolerance (Fig. 9).

## Discussion

### Dietary *E. coli* are a major source of Moco for *C. elegans*

The free-living nematode *C. elegans* acquires Moco from its bacterial diet or by synthesizing it *de novo* from GTP. Under standard laboratory conditions, either source of Moco is sufficient to promote growth, development, and reproduction. Thus, when growth conditions are ideal, these pathways appear to operate redundantly to support life. However, this conclusion relies exclusively on measuring the rate of development and fertility (14).

To expand our understanding of Moco homeostasis, we produced *C. elegans* extracts from animals lacking either endogenous Moco synthesis or dietary Moco. These extracts were used to quantify Moco content and SUOX-1 activity, providing biochemical clarity to our genetic system (12, 13, 14). We found that *C. elegans* mutants lacking endogenous Moco biosynthesis displayed reduced Moco content and SUOX-1 activity. *moc-4* and *moc-6* mutant *C. elegans* fed Moco+ *E. coli* showed 64% and 65% of Moco content and 28% and 39% SUOX-1 activity, respectively, compared to WT controls. These decreases were expected as Moco synthesis has long been understood to be an essential source of Moco for the cell.

Surprisingly, we found even more severe defects when evaluating the biochemical impacts of a Moco-deficient diet. WT *C. elegans* fed a Moco- diet displayed 18% of the Moco content and 11% of SUOX-1 activity compared to WT animals fed a Moco+ diet. This demonstrates that the *C. elegans* diet substantially contributes to Moco homeostasis in *C. elegans* animals. Thus, dietary Moco and endogenous Moco synthesis function nonredundantly to promote Moco content and SUOX-1 activity.

Having demonstrated the biochemical impacts of either dietary or endogenous Moco loss, we wondered whether these deficiencies in Moco content or SUOX-1 activity would cause a fitness defect. To evaluate this, we employed sulfite as a pharmacological intervention. Sulfite is a useful tool as it is a toxin and the primary substrate of SUOX-1. Sulfite is typically produced as a byproduct of sulfur amino acid metabolism (25). Thus, maintaining SUOX-1 activity is critical for sulfite detoxification and *C. elegans* survival. We observed that *C. elegans* lacking either source of Moco displayed sulfite sensitivity, demonstrating the nonredundant roles of dietary and endogenous Moco in promoting sulfite tolerance. Supporting this result, we found that both sources of Moco were also required for life in a *suox-1(gk738847)* hypomorphic mutant background.

Given these results, we propose the model that when food is abundant, *C. elegans* animals rely on both endogenous Moco synthesis and dietary Moco to support Moco homeostasis (Fig. 9). While endogenous Moco synthesis has long been established to be critical for supporting Moco homeostasis, our work demonstrates the equal importance of dietary Moco in the life and survival of *C. elegans.*

### Are two sources of Moco better than one?

Why would the *C. elegans* genome retain Moco biosynthetic machinery if the animals can acquire the cofactor so efficiently from the diet? Why is Moco not a vitamin for *C. elegans*, as is the case for other essential coenzymes (26, 27, 28)? Logic dictates that there must be a fitness advantage to maintaining both strategies of increasing cellular Moco. We speculate that acquiring Moco from dietary microbes (when they are abundant) is energetically favorable compared to *de novo* Moco synthesis from GTP. However, endogenous Moco synthesis is likely a more reliable Moco source during the life of a wild *C. elegans* individual. This is because wild *C. elegans* have a “boom-and-bust” life cycle (29). When conditions are ideal, newly hatched *C. elegans* pass through four larval stages and reach fertile adulthood in about 3 days. However, under stressful conditions (low food, high population density, high temperature), *C. elegans* enter an alternative nonfeeding diapause state known as dauer (30). Dauer larvae are stress resistant and can survive months as they disperse and seek new food. Wild *C. elegans* live the majority of their lives as nonfeeding dauer larvae that are likely reliant on endogenous Moco synthesis (31). As dauer larvae locate food (usually microbes found on rotting fruit or plant stems) and return to favorable conditions, they resume development and reach fertile adulthood (30). During this “boom” time, dietary Moco would be abundant as ∼70% of bacterial genomes encode Moco biosynthetic enzymes (2). Yet, the food source will eventually be consumed, dauer larvae will emerge in the population, and the cycle will repeat. Considering this natural history, redundant sources of Moco seem logical. When no food is available for weeks/months, the animals must rely on endogenous Moco synthesis to survive. However, once a microbial food source is identified, *C. elegans* additionally harvest Moco from their diet, promoting Moco accumulation and enzymatic function within the animals.

### Moco bioavailability in *C. elegans* and beyond

Moco is an ancient and essential prosthetic group; Moco synthesizing and requiring enzymes were present in the last universal common ancestor and persist in all domains of life today (1, 2). Thus, it seems likely that Moco biology uncovered in the nematode *C. elegans* will be found throughout the tree of life. Our discovery that dietary Moco promotes Moco content, SUOX-1 activity, and rescues *C. elegans* Moco deficiency lays additional intellectual groundwork for developing Moco supplementation as a therapy to treat human Moco deficiency (13, 14). However, uptake of exogenous Moco has yet to be examined or observed in another organism. It is crucial to determine if human cells are competent to acquire exogenous Moco as this is a critical next step in developing therapeutic Moco.

In addition to therapeutic considerations, insights into Moco biology from the nematode *C. elegans* reveal many new fundamental questions about Moco biology: how is Moco moving from bacteria to *C. elegans*? How is Moco stably harvested from the exogenous donor proteins? Once harvested, how is Moco traversing cell membranes? Are there animal Moco chaperones facilitating these processes? Given the ancient and conserved nature of Moco, we expect pathways and phenomena uncovered in *C. elegans* to be at play across the diversity of life.

## Experimental procedures

### Animal cultivation

*C. elegans* strains were cultured using established protocols (32). Briefly, animals were cultured at 20 °C on nematode growth media (NGM) seeded with WT *E. coli* (OP50) unless otherwise noted. The WT *C. elegans* strain was Bristol N2. All mutant alleles and transgenes used in this study have been previously published.

Additional *C. elegans* strains used in this work and their origins are described here:

GR2253 [*moc-4(ok2571) I*] (14),

FX30167 [*tmC18 [dpy-5(tmIs1200)] I*] (24),

USD989 [*moc-4(ok2571) I; cdo-1(mg622) X*] (engineered for this study),

USD1011 [*moc-4(ok2571)/tmC18 [tmIs1236] I; suox-1(gk738847) X*] (engineered for this study),

USD955 [*moc-6(rae296) III*] (12),

USD959 [*moc-6(rae296) III; cdo-1(mg622) X*] (12),

GR2256 [*moc-5(mg589) X*] (14),

GR2254 [*moc-1(ok366) X*] (14),

GR2260 [*cdo-1(mg622) X*] (14), and

GR2269 [*suox-1(gk738847) X*] (14).

To generate strains USD989 and USD1011, animals were mated using standard *C. elegans* husbandry and critical genetic loci were monitored as described here. The *moc-4(ok2571)* deletion was monitored by PCR using the following primers: 5′-gcagttatgaggcgaaggag-3′, 5′-tcgagcccactctttctctg-3′, and 5′-cgtcacacgagaatgcaaga-3’ (14). The *cdo-1(mg622)* single nucleotide polymorphism was monitored by PCR amplification and Sanger sequencing using the following primers: 5′-gcaaatgagtggcgaagatt-3′ and 5′-cgccgattgactaacctcat-3’ (14). The *tmC18 [dpy-5(tmIs1200)]* balancer was monitored by the expression of the dominant *Pmyo-2::Venus* fluorescent transgene in combination with a recessive dumpy (Dpy) phenotype caused by *dpy-5(tmIs1200)* (24)*.*

Additional *E. coli* strains used in this work were as follows:

BW25113 (WT, Moco+) and

JW0764-2 (*ΔmoaA753::kan,* Moco-).

### Protein and metabolite extraction from *C. elegans*

Synchronized populations of 10,000 to 20,000 *C. elegans* animals were cultured at 20 °C and harvested at the young adult stage of development. To remove dietary bacterial cells from the sample, animals were washed in excess M9 buffer three times and then incubated in M9 buffer for 1 h at 20 °C with gentle rocking. Animals were subsequently washed one additional time with excess M9 buffer, pelleted, and flash frozen in liquid nitrogen. All samples were cultured on either BW25113 (WT, Moco+) or JW0764-2 (*ΔmoaA753::kan,* Moco-) *E. coli* for the entire experiment. However, if *moc-*mutant *C. elegans* are cultured on *ΔmoaA* (Moco-) *E. coli* from early larval stages, they undergo larval arrest and death (12, 14). To allow for biochemical analyses of *moc-4* and *moc-6* single mutant animals cultured on *ΔmoaA* (Moco-) *E. coli*, these mutant strains were initially cultured on Moco+ *E. coli* until the L4 stage of development (48 h post L1 synchronization). These L4 larvae were then collected, extensively washed as described above, and reintroduced onto new culture dishes containing NGM seeded with Moco- *E. coli.* Thus, dietary Moco was depleted at a later stage in development, as previously described (Table S1) (12, 14).

For total protein and metabolite extraction, flash-frozen *C. elegans* samples were lysed in 400 μl of lysis buffer (20 mM Hepes, 150 mM NaCl, 1 mM EDTA, 0.5% Triton X-100, pH 7.5) using a FastPrep-24 (M.P. Biomedicals Irvine) four times for 30 s at 6.5 m/s with 5 min breaks and then incubated for 30 min on ice. Protein concentrations were determined using a Bradford protein assay (Carl Roth Karlsruhe). Absorption was measured using a Multiskan GO microplate spectrophotometer (ThermoFisher Scientific).

### Detection of sulfite oxidase activity

Sulfite oxidase activity measurements were based on a sulfite-dependent enzymatic reduction of cytochrome c (19). Ten to fifty micrograms protein of total crude extract from *C. elegans* were used for quantification. Fifty microliters of each dilution were loaded into a 96-well plate. One hundred eighty microliters of the SUOX-1 activity buffer (100 mM Tris–HCl, 0.1 mM EDTA, 0.04 mM cytochrome c_ox_, pH 8.5) were added to the well. Subsequently, samples were incubated for 5 min on ice. The reaction was initiated by adding 20 μl of a 5 mM sodium sulfite solution. As a control, 20 μl _dd_H_2_O was added instead. Immediately after adding sulfite, an absorption change at 550 nm was measured in a Multiskan GO microplate spectrophotometer (ThermoFisher Scientific). The detection was set over a time course of 60 min with 720 measurements. For visualization, the _dd_H_2_O control was subtracted from the sample with sulfite, which was used to plot a time-dependent absorbance change. The SUOX-1 activity was determined from the slope of the linear range of the curve. 1U is defined as the amount of protein required to reach an absorption increase of 1.0 per minute.

### Quantification of Moco/MPT using FormA

Quantifying the Moco and MPT content of crude *C. elegans* extracts was performed by oxidizing Moco/MPT in a well-defined environment to FormA, a stable and fluorescent Moco/MPT oxidation product (10). For oxidation of Moco, 500 μg of crude protein extract in 400 μl of 100 mM Tris–HCL pH 7.2 were used. Samples were oxidized with 50 μl oxidation solution (1% I_2_, 2% KI in 1 M HCl) for 16 h in the dark at room temperature (RT). After oxidation, the samples were centrifuged at 10,000*g* for 10 min, and the supernatant was transferred to a new reaction tube. Fifty-five microliters of 1% ascorbic acid solution were added to stop the oxidation reaction. Subsequently, 200 μl 1 M Tris, 13 μl MgCl_2_, and 2 μl alkaline phosphatase (Roche Basel) were added and incubated overnight in the dark at RT. The HPLC detection of _dp_FormA was performed at RT using a reversed-phase C-18 column (250 mm × 4.6 mm, 5 μm, ReproSil-Pur Basic C-18 HD) with an Agilent 1100 HPLC system containing a fluorescence detector (20). The specific parameters were set with a flow rate of 1 ml min^−1^ using an isocratic run with 5 mM ammonium acetate and 15% (v/v) methanol as the mobile phase. For the fluorescence detection λ ex = 302 nm, λ em = 451 nm were set. For data analysis, OpenLab CDS Version 2.2.0.600 was utilized. Calibration was carried out using synthetic _dp_FormA (21).

To monitor _dp_FormA content in *E. coli*, bacterial strains were cultivated by inoculation from glycerol stocks in 50 ml LB medium and grown overnight at 37 °C in a shaking incubator. Bacteria were centrifuged at 10,000*g* for 10 min at 4 °C. Subsequently, the supernatant was discarded, and the pellet was resuspended with 25 ml chilled 100 mM Tris–HCI (pH 7.2). After this washing step, the cells were centrifuged again at 10,000*g* for 10 min at 4 °C and the supernatant was discarded. The cell pellets were resuspended with 1.5 ml 100 mM Tris–HCI (pH 7.2). Cell lysis and FormA oxidation were performed as described for *C. elegans*, except we used 0.1 mm glass beads to disrupt the bacteria.

### *C. elegans* growth assays

Growth assays were performed as previously described with minor modifications (13, 14). Briefly, WT and mutant *C. elegans* were synchronized at the L1 stage. To synchronize animals, embryos were harvested from gravid adult mothers *via* treatment with a bleach and sodium hydroxide solution (33). Embryos developed overnight in M9 solution causing them to hatch and arrest development at the L1 stage. Subsequently, animals were cultured on NGM seeded with WT or mutant *E. coli* for 72 h at 20 °C. Live animals were then imaged using an SMZ25 stereomicroscope (Nikon) equipped with an ORCA-Flash4.0 camera (Hamamatsu). Images were captured using NIS-Elements software (Nikon) and processed using ImageJ (NIH). Animal length was measured from the tip of the head to the end of the tail.

For sulfite sensitivity experiments, the dietary *E. coli* were pelleted and resuspended with various concentrations of sodium sulfite (Millipore Sigma) in water. The *E. coli* (5× concentrated)*-*sulfite slurries were then seeded onto the NGM media as a food source for *C. elegans.* The concentration of sulfite displayed assumes the sulfite diffuses evenly throughout the 10 ml of NGM media. A limitation to this method is that sulfite oxidizes to sulfate in water, and thus, the presented concentrations likely overestimate the sulfite concentration experienced by *C. elegans* in these assays (34, 35). To mitigate sulfite oxidation, we worked quickly to limit the time between establishment of the culture dishes (NGM, *E. coli*, sulfite) and the addition of the appropriate *C. elegans* animals. Growth assays were then performed as described previously.

For experiments where Moco+ and Moco- bacterial diets were mixed, BW25113 and JW0764-2 *E. coli* were first cultured overnight in LB at 37 °C in a shaking incubator. Overnight cultures were then concentrated 10 times. These 10× concentrated stocks were then mixed yielding various fractions of Moco+ (WT) *E. coli* in Moco- (*ΔmoaA*) mutant *E. coli*. These *E. coli* mixtures were then seeded onto NGM supplemented with the antibiotic Streptomycin, preventing additional *E. coli* growth. Growth assays were performed as described previously.

### Statistics

Welch’s unpaired *t* test or Ordinary One-Way ANOVA was used to determine significant differences between two or more than two groups. Dunnet’s post hoc analysis was employed to determine significant differences between groups when compared to a reference group. A Tukey’s post hoc test was used to determine significance between all comparisons. The analyses used are indicated in the appropriate figure legends. A *p*-value < 0.05 was considered statistically significant for all analyses. To characterize dose-response curves, nonlinear regression with IC_50_ analyses were utilized and incorporated experimentally derived minimum and maximum animal length constraints. 95% confidence intervals are displayed in shading along each regression curve presented in the figures. R-squared values for each curve are presented in the appropriate figure panels. All analyses were completed using GraphPad Prism software (GraphPad Software Inc).

## Data availability

All data are presented within the article.

## Supporting information

This article contains supporting information.

## Conflict of interest

The authors declare that they have no conflicts of interest with the contents of this article.
