# Supplementary material for: Obtaining the necessary molybdenum cofactor for sulfite oxidase activity in the nematode Caenorhabditis elegans surprisingly involves a dietary source
Source: J Biol Chem. 2022 Nov 22;299(1):102736. doi: 10.1016/j.jbc.2022.102736 (PMC9793310; doi:10.1016/j.jbc.2022.102736)
Supplement: Supporting information [file mmc1.pdf]

Supporting Information for:

Obtaining the necessary molybdenum cofactor for sulfite oxidase activity in the nematode *C. elegans* surprisingly involves a dietary source

Kevin D. Oliphant[1+], Robin R. Fettig[2,4+], Jennifer Snoozy[2+], Ralf R. Mendel[1], Kurt Warnhoff[2,3]

[1]Department of Plant Biology, Braunschweig University of Technology, 38106 Braunschweig, Germany
[2]Pediatrics and Rare Diseases Group, Sanford Research, Sioux Falls, SD 57104, USA
[3]Department of Pediatrics, Sanford School of Medicine, University of South Dakota, Sioux Falls, SD 57105, USA
[4]Department of Basic Biomedical Sciences, Sanford School of Medicine, University of South Dakota, Vermillion, SD 57069, USA

*Corresponding author: Kurt Warnhoff, kurt.warnhoff@sanfordhealth.org
[+]These authors contributed equally to this work.

This Supporting Information file contains:

Supporting Figure 1
Supporting Figure 2
Supporting Figure 3
Supporting Table 1

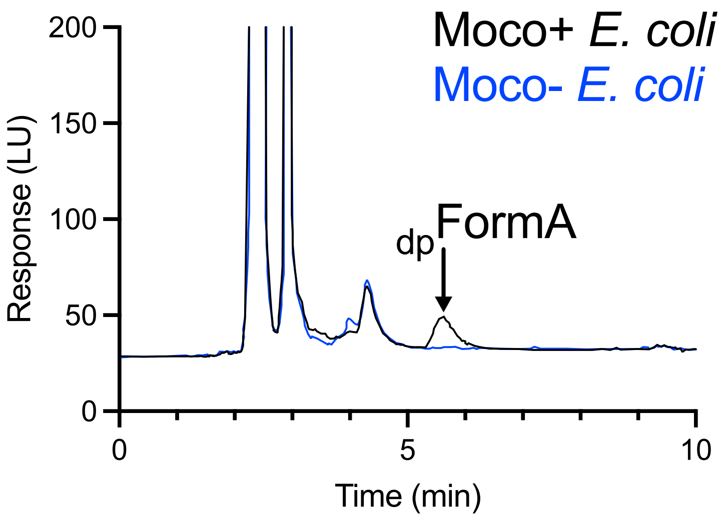

Supporting Figure 1: $_{dp}$FormA is not detected in *ΔmoaA* mutant *E. coli.*
HPLC measurements of Moco-derived dephospho-FormA ($_{dp}$FormA) from extracts of wild-type (black, Moco+) and *ΔmoaA* mutant (blue, Moco-) *E. coli*. The $_{dp}$FormA peak is indicated (black arrow).

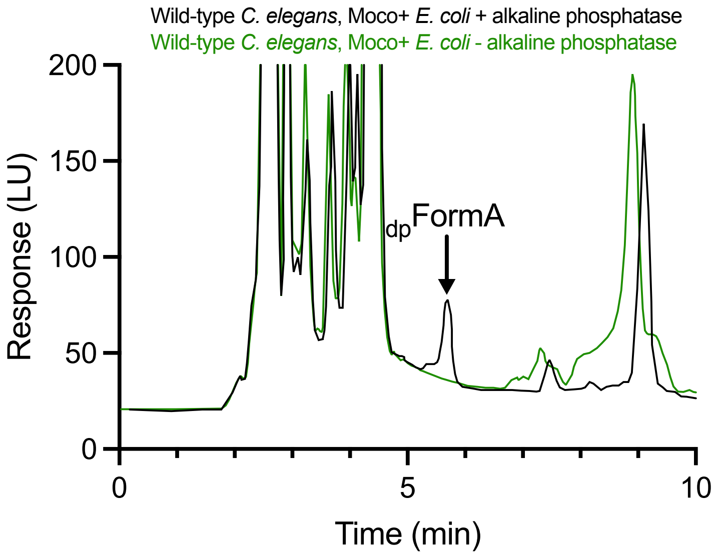

Supporting Figure 2: FormA analysis with and without the addition of alkaline phosphatase.
HPLC measurements of Moco-derived dephospho-FormA ($_{dp}$FormA) from crude extracts of wild-type *C. elegans* fed wild-type (Moco+) *E. coli*. Oxidized extracts were evaluated with (black) or without (green) alkaline phosphatase treatment. The $_{dp}$FormA peak is indicated (black arrow).

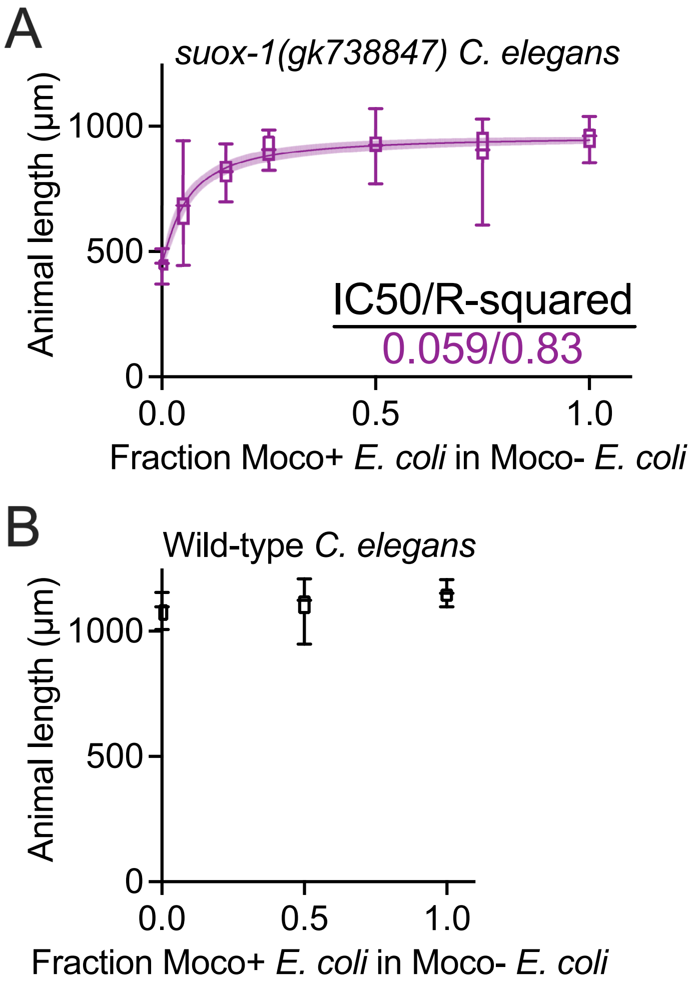

Supporting Figure 3: *suox-1(gk738847)* mutant *C. elegans* are sensitive to dietary Moco deficiency.
A) *suox-1(gk738847)* mutant and B) wild-type *C. elegans* were synchronized at the L1 stage and cultured on diets with different fractions of wild-type (Moco+) in *ΔmoaA* mutant (Moco-) *E. coli* (0, 0.05, 0.15, 0.25, 0.5, 0.75, 1). Animal length was measured after 72 hours of growth at 20°C. Sample size is 15 individuals per data point. Box plots display the median, upper, and lower quartiles, while whiskers indicate minimum and maximum data points. For panel A, IC_{50} was calculated by non-linear regression analysis and shading indicates the 95% confidence interval. IC_{50} and R-squared values are displayed. Note, datapoints at 0, 0.5, and 1.0 fraction Moco+ in Moco- *E. coli* for *suox-1* mutant *C. elegans* are also displayed in Fig. 7A.

C. elegans genotype	Diet	SUOX-1 activity ± standard deviation (U/mg)	SUOX-1 activity sample size	dpFormA ± standard deviation (pmol/mg)	dpFormA sample size	Figures in which data displayed
Wild type	Moco+	0.33±0.05	10	1.31±0.3	10	2C, 3C, 4A, 4B, 5A, 5B, 6A
	Moco-	0.035±0.009	6	0.23±0.1	6	4A, 4B
moc-4(ok2571)	Moco+	0.091±0.002	3	0.84±0.3	3	5A, 5B
	Moco-*	0.033±0.01	3	0.24±0.02	3	
moc-4(ok2571); cdo-1(mg622)	Moco+	0.11±0.02	3	1.10±0.4	3	
	Moco-	None detected	3	None detected	3	2C, 3C
moc-6(rae296)	Moco+	0.13±0.02	9	0.85±0.2	5	5A, 5B
	Moco-*	0.021±0.006	6	0.29±0.08	3	
moc-6(rae296); cdo-1(mg622)	Moco+	0.13±0.01	6	1.35±0.4	6	
	Moco-	None detected	6	None detected	6	2C, 3C
suox-1(gk738847)	Moco+	0.013±0.001	3	Not measured	Not measured	6A

Supporting Table 1: Biochemical analyses of SUOX-1 activity and $_{dp}$FormA content in *C. elegans.*

SUOX-1 activity and dephospho-FormA ($_{dp}$FormA) content are displayed for wild-type and mutant *C. elegans* fed wild-type (Moco+) or *ΔmoaA* mutant (Moco-) *E. coli.* Average, standard deviation, and sample size (biological replicates) are displayed for each condition. The figure panels where each dataset are presented are also highlighted for SUOX-1 activity (red) or $_{dp}$FormA content (blue). *, animals were shifted to Moco- *E. coli* at the L4 stage to permit larval development (see Experimental procedures).
